# The Mechanisms of Systemic Inflammatory and Immunosuppressive Acute-on-Chronic Liver Failure and Application Prospect of Single-Cell Sequencing

**DOI:** 10.1155/2022/5091275

**Published:** 2022-11-02

**Authors:** Kai Kou, Xiaodong Sun, Guangyao Tian, Yao Zhi, Zhongqi Fan, Guoyue Lv

**Affiliations:** Department of Hepatobiliary and Pancreatic Surgery, The First Hospital of Jilin University, Changchun, 130021 Jilin, China

## Abstract

Acute-on-chronic liver failure (ACLF) is a complex clinical syndrome, and patients often have high short-term mortality. It occurs with intense systemic inflammation, often accompanied by a proinflammatory event (such as infection or alcoholic hepatitis), and is closely related to single or multiple organ failure. Liver inflammation begins when innate immune cells (such as Kupffer cells (KCs)) are activated by binding of pathogen-associated molecular patterns (PAMPs) from pathogenic microorganisms or damage-associated molecular patterns (DAMPs) of host origin to their pattern recognition receptors (PRRs). Activated KCs can secrete inflammatory factors as well as chemokines and recruit bone marrow-derived cells such as neutrophils and monocytes to the liver to enhance the inflammatory process. Bacterial translocation may contribute to ACLF when there are no obvious precipitating events. Immunometabolism plays an important role in the process (including mitochondrial dysfunction, amino acid metabolism, and lipid metabolism). The late stage of ACLF is mainly characterized by immunosuppression. In this process, the dysfunction of monocyte and macrophage is reflected in the downregulation of HLA-DR and upregulation of MER tyrosine kinase (MERTK), which weakens the antigen presentation function and reduces the secretion of inflammatory cytokines. We also describe the specific function of bacterial translocation and the gut–liver axis in the process of ACLF. Finally, we also describe the transcriptomics in HBV-ACLF and the recent progress of single-cell RNA sequencing as well as its potential application in the study of ACLF in the future, in order to gain a deeper understanding of ACLF in terms of single-cell gene expression.

## 1. Introduction

The liver plays an important role in daily immune monitoring [1]. Due to its special blood supply characteristics, it is constantly exposed to host antigens and various pathogens and toxins from the intestine [2]. In a healthy state, Kupffer cells (KCs), the main phagocytic cells of the liver, account for more than 80% of the total macrophages. Along with dendritic cells (DCs) and neutrophils, KCs mount the innate immune response and play a critical role in the adaptive immune response [3, 4]. In case of injury, the hepatic macrophage counts increase through recruiting monocytes from the bone marrow. In acute-on-chronic liver failure (ACLF), macrophage-mediated inflammation may progress to systemic inflammation and subsequent immunosuppression [5, 6]. An excessive systemic inflammatory response leads to organ failure and death, and immunosuppression makes patients prone to secondary infection events, further exacerbates organ dysfunction, and increases mortality [7]. Based on the latest discovery and research of ACLF, we summarized the clinical manifestations and immunological and microbiological roles in the pathogenesis of ACLF. In addition, we also discussed the preliminary application and application prospect of sequencing technology in ACLF.

## 2. Clinical Features

Currently, there are two commonly used definitions of ACLF. One is the definition of the European Association for the Study of the Liver-Chronic Liver Failure Consortium (EASL-CLIF ACLF): (1) patients with acute decompensated cirrhosis have previous episodes of decompensation; (2) precipitating events include intrahepatic (alcoholic hepatitis), extrahepatic (infection, gastrointestinal bleeding, and drug-induced encephalopathy), or both; (3) organ dysfunction involves one or more of the six organ systems (liver, kidney, brain, coagulation, circulation, and respiration); and (4) short-term mortality rate is high (20-80% at 28 days)[8–12]. The other is the definition of Asian Pacific Association for the Study of the Liver (APASL ACLF): patients with compensatory cirrhosis (diagnosed or undiagnosed) or noncirrhotic chronic liver disease have the first episode of acute liver deterioration due to acute liver injury directly caused by intrahepatic precipitating events and which involves liver dysfunction, which eventually causes encephalopathy and high short-term mortality (13-86% at 30 days) [13]. For the convenience of subsequent discussion, we combined these two definitions and defined ACLF as a syndrome of acute liver deterioration induced by precipitating events in patients with cirrhosis or noncirrhotic chronic liver disease, leading to failure of two or more organs, accompanied by short-term high mortality.

A number of factors can contribute to ACLF precipitating events. Alcoholic cirrhosis is very common in ACLF in western countries. In Asian countries, however, hepatitis B is a more common precipitating event in patients with ACLF [14]. The infection rate of ACLF patients is extremely high (66.1%). In contrast, acute decompensation (AD) patients have an overall infection rate of 38.7% [15]. Disease progression in ACLF patients is primarily caused by gram-negative bacterial infection, exacerbated by bacterial translocation [5]. In addition, age, mitochondrial damage, and decreased sex hormones may lead to the premature occurrence of immunosenescence and inflammation in chronic liver disease, increasing the risk of ACLF [7].

ACLF has a high short-term mortality rate. Mortality in ACLF patients is higher than in patients with decompensated chronic liver disease, and the number of organ failures rather than the etiology or predisposing event of cirrhosis is the main risk factor for death [16]. Mortality at 28 and 90 days in cirrhosis patients with AD is 5% and 14%, respectively, while in ACLF, it is between 22-78% and 41-79% depending on the grade [8, 17].

## 3. The Initiation of Hepatic Inflammation

ACLF is usually initiated by hepatic inflammation mediated by inflammatory factors. Inflammatory factors in ACLF patients are exogenous or endogenous [18]. The exogenous factors are mainly bacteria, HBV, and alcohol. The innate immune system initiates its response to invading pathogens upon the recognition of pathogen-associated molecular patterns (PAMPs) by pathogen recognition receptors (PRRs). Peripheral blood mononuclear cells (PBMCs) isolated from cirrhotic patients responded more strongly when stimulated by lipopolysaccharide (LPS) than PBMCs from healthy patients [19]. This demonstrates that the pathogen recognition function of the innate immune system is activated in cirrhotic patients, which is more conducive to triggering liver inflammation.

Endogenous factors in ACLF patients include the release of necrotic cells or extracellular matrix degradation [20]. Sterile inflammation results from the recognition of damage-associated molecular patterns (DAMPs) after tissue injury [3, 21]. DAMPs are usually sequestered intracellularly, and after being released extracellularly, binding by PRRs on immune cells then triggers an inflammatory response, which leads to the activation of immune cells and a kind of proinflammatory phenotype, thereby initiating inflammatory signals through the release of cytokines and chemokines, which in turn aggravates the inflammatory response in ACLF [22].

## 4. Systemic Inflammation in ACLF

The persistence of hepatic inflammation can develop into systemic inflammation. Patients with decompensated cirrhosis develop persistent systemic inflammation because of gut dysbiosis, disruption of intestinal mucosal barrier integrity, and persistent translocation of PAMPs [23]. Systemic inflammation drives the occurrence and development of ACLF and causes extensive tissue and organ damage, which leads to systemic inflammatory response syndrome (SIRS) [24, 25]. In order to overcome SIRS, the body develops compensatory anti-inflammatory responses (CARS), which in turn promote the occurrence of infection and aggravate the proinflammatory response [26].

Macrophages are highly diverse and plastic and play a leading role in the development of ACLF. They have important functions in the response to injury or infection [27]. In the early stage of liver injury, liver macrophages secrete both proinflammatory and anti-inflammatory cytokines to mediate proinflammatory and anti-inflammatory responses [28]. Disruption of the intestinal barrier in ACLF patients leads to the translocation of PAMPs such as intestinal bacteria and their products to the liver, which activates macrophages through Toll-like receptors (TLRs), resulting in the secretion of a large number of cytokines and the recruitment of various immune cells, leading to liver and systemic inflammation [23, 29].

During this process, hepatic macrophages can quickly change their phenotype according to the local microenvironment of the liver [30]. They are traditionally divided into M1 and M2 macrophages, according to their differentiation state [31]. The two macrophage populations are functionally distinct: typical functions of the M1 macrophages include antigen presentation and secretion of cytokines IL-6, IL-12, TNF-*α*, IL-1, CXCL1-3, CXCL-5, CXCL8-10, and type I IFN, reactive oxygen species (ROS), and nitric oxide, which help in inflammatory [32]. In contrast, M2 macrophages show a resting phenotype, expressing mannose receptors, scavenger receptors A and B-1, and CCR2 and CD163, which are involved in tissue healing [33]. CD163, TGM2, and CD206 levels are increased in ACLF patients [26]. IL-4, IL-10, and IL-13 are typically produced by M2 macrophages [34]. It has been reported that macrophages release TNF-*α* and IL-6 in the initial stage of ACLF, followed by IL-10 [35].

In addition to these resident KCs, liver-recruited monocytes also play an anti-inflammatory or proinflammatory role at various stages of the disease [36]. KCs are exclusively intravascular and distributed along hepatic sinusoidal endothelial cells (HSECs), while monocyte-derived macrophages (MoMFs) and monocytes could reside outside the blood vessels [37]. This distribution is beneficial for monocyte and macrophage to play a more comprehensive immune regulatory role in systemic inflammation of ACLF.

## 5. Mediators of Inflammation in ACLF

Cytokines play an important regulatory role in ACLF. Cytokines are glycoproteins that regulate innate immunity by inducing local and systemic inflammatory responses in ACLF [38]. Activated immune cells secrete a variety of cytokines that further promote tissue damage [14]. Both proinflammatory molecules and anti-inflammatory factors are enhanced during ACLF, reflecting the general activation of cytokine cascades [24, 39].

In ACLF, cytokines are mainly divided into proinflammatory cytokines, including TNF-*α*, IL-1*β*, and IL-6, and anti-inflammatory cytokines, such as IL-10, IL-4, and IL-1 receptor antagonists [40]. On the one hand, IL-1*β*, LPS, or TNF-*α* stimulates TLR4 to induce the synthesis and secretion of IL-6, which is one of the main stimulators of acute phase protein release. The TNF-*α* signaling pathway, driving apoptosis and necrosis, may be involved in the occurrence of liver injury during ACLF [26]. On the other hand, the level of IL-10 secreted by monocytes in the early stage of ACLF patients is lower than that of healthy controls, but IL-10 secretion is increased in late periods of ACLF [41].

In addition, growth factors such as granulocyte-macrophage colony-stimulating factor (GM-CSF) and granulocyte colony-stimulating factor (G-CSF) are involved in the hematopoiesis and proliferation of liver progenitor cells [42]. In fact, G-CSF improves liver function and survival in ACLF [43]. G-CSF amplifies circulating myeloid and plasmacytoid DCs (mDCs, pDCs) and T cells but decreases IFN-*γ* production in CD8^+^T cells [44]. In ACLF patients, dysregulation of IFN-*γ* causes systemic inflammation and impaired liver regeneration [45].

## 6. Systemic Immunosuppression in ACLF

ACLF is not only associated with severe systemic inflammation, but as the disease progresses, it is also associated with immune tolerance, an adaptive response that reduces the adverse effects of damage on the host [46]. Upon innate immune paralysis, proinflammatory immune cells and proinflammatory factor decrease, and inhibitory immune cells and anti-inflammatory substances increase [47, 48]. In ACLF, tissue macrophages exhibit endotoxin tolerance/immunomodulatory functions. These cells circulate through the bloodstream and further spread to other tissues, thereby contributing to the immunosuppressive phenotype of ACLF [48]. Immune dysfunction leads to the prevalence of infections and low survival in ACLF [24].

The development of immunosuppressive in ACLF involves multiple systems, such as the circulatory, intestinal, hepatic, peritoneal (spontaneous bacterial peritonitis), and reticuloendothelial (RES) systems [49]. Among them, RES-mediated clearance of pathogens depends on the severity of liver dysfunction and downregulates the bactericidal capacity of phagocytes by reducing the synthesis of innate antibacterial proteins in the liver [50]. With the development of the disease, some phenotypic changes of immune cells play a major role in the systemic immunosuppression of ACLF, including innate immune cells and adaptive immune cells, in which the phenotypic changes of monocyte and macrophage play the most prominent role.

### 6.1. Defects in Innate Immune Cells of ACLF

#### 6.1.1. Monocyte and Macrophage

The expression of HLA-DR reflects the immune response function of monocyte and macrophage. In the early stages of the disease, IL-33 activates the ERK1/2 pathway to restore the expression of HLA-DR, CD80, and chemokine receptor 2 in monocytes and enhances the expression of proinflammatory cytokines in monocytes without affecting their phagocytic activity [51]. In the late stages of the disease, monocyte and macrophage defects in ACLF patients include decreased immune response to microorganism and dysfunction of antigen presentation through decreasing the HLA-DR expression [48, 52]. Although the reduced innate response is a physiological adaptation to continuous PAMP exposure, this change in turn aggravates secondary infection and leads to higher mortality [48, 50]. Moreover, the low HLA-DR expression is positively correlated with prothrombin time, an indicator of liver injury [41].

Another important phenotypic change in immunosuppression is MER tyrosine kinase (MERTK). The upregulation of MERTK on monocytes can cause the immune dysfunction of ACLF [48]. MERTK inhibits inflammation by activating inhibitors of cytokine signaling, blocking TLR activation, and decreasing proinflammatory cytokine production [48]. The MERTK overexpression reduces the in vitro response to LPS and is strongly correlated with ACLF immunosuppression, the SIRS activation level, and disease severity [48].

The amplification of monocytic myeloid-derived suppressor cells (M-MDSCs) and intermediate CD14^+^CD16^−^ monocytes also plays an important role in ACLF immunosuppression [53, 54]. In ACLF, the expression of M-MDSCs is immunosuppressed by decreasing T cell proliferation, TNF-*α* secretion, and the phagocytosis of *Escherichia coli*. Immunosuppression of M-MDSCs may contribute to infection, while TLR3 activation could reverse the expansion of these cells and restore the function of innate immune [53]. MDSCs are also closely associated with the MELD score. In advanced ACLF, nonsurvivors maintain the highest numbers of MDSCs, while survivors show a gradual decline [55]. The intermediate CD14^+^CD16^−^ monocyte subpopulation is featured by the production of fewer proinflammatory cytokines and more IL-10 after stimulation [48, 53]. Transcriptional profiling also revealed that immunosuppressive parameters are enhanced and antibacterial and antigen presentation mechanisms are impaired [26]. Functional alterations in classical CD14^+^CD16^−^ monocytes are also evident in ACLF patients, and genes related with immunosuppressive responses are upregulated. Glutamine synthase inhibitors can partially restore the phagocytosis of ACLF monocytes [54].

In addition, hypoxia-inducible factor 1 alpha-antisense RNA 1 (HIF1A-AS1) also plays a role in the dysfunction of monocyte and macrophage. Studies have shown that TNF-*α* promotes KC apoptosis by inducing the expression of HIF1A-AS1 [56]. And the apoptosis of KCs may increase the chance of exposure to DAMPs and persistent bacteremia [5].

#### 6.1.2. Neutrophils

Neutrophil dysfunction is associated with secondary infection, organ failure, and high mortality [57–59]. Neutrophils secrete large amounts of neutrophil gelatinase-associated lipocalin (NGAL), also named lipidcalin-2, which plays an essential role in innate immunity [60]. ACLF patients had higher basal ROS levels in neutrophils at the early stage, suggesting a neutrophil priming state, whereas fMet-Leu-Phe- (fMLP-) stimulated ROS production is reduced [61, 62]. The reduction in ROS production is due to a marked reduction in phospholipase C activity [63], insufficient phosphorylation [64], and a decrease in protein expression [62]. Together with a defect in the extracellular function of myeloperoxidase that may be the result of reduced AKT and p38 MAP kinase activation, downregulated ROS generation results in insufficient bactericidal activity in late stage of ACLF [64, 65].

In addition, CD11b^+^CD16^+^ neutrophils from ACLF patients also overexpress the chemokine receptors CXCR1 and CXCR2, which recognize IL-8, and then induce hepatocyte death [61]. It has also been reported that CXCR1/2 regulates the secretion of various kinds of mediators, causing oxidative stress, which in turn induces cell death [26, 61].

#### 6.1.3. DCs

ACLF expresses low levels of mDCs and pDCs, and even lower levels of these cells are found in nonsurviving patients. Low DC counts are strongly correlated with high mortality. The number of DCs could be enhanced by G-CSF [66]. Treatment with methylprednisolone resulted in increased DC counts, improved liver function, and reduced mortality [67]. In ACLF, monocyte-derived dendritic cells (MoDCs) secrete large amounts of IL-23 and express their receptor IL-23R. Elevated IL-23 levels in nonsurvivors suggest that IL-23 is associated with disease progression and severity [68].

#### 6.1.4. NK Cells

NK cells make up about 15% of lymphocytes in the blood, increasing to 30% in the liver. Even in the absence of MHC, NK cells can recognize damaged cells, which results in a faster immune response [26]. The number of NK cells and CD56^dim^ CD16^bright^ NK cells decreases in hepatitis B virus-associated ACLF (HBV-ACLF) [69]. NK cell function is regulated through activating cytotoxicity and inhibiting receptors. In ACLF patients, in addition to downregulation of CD158b, both activating and inhibitory receptors are upregulated. NK cell-mediated killing is significantly reduced in HBV-ACLF, as well as TNF-*α* production and cytotoxic activity [69, 70], suggesting that inhibitory receptors are superior to activating receptors.

In addition, the increase of CD57^+^ CD3^+^ NK cells in the liver leads to hepatocyte necrosis and leads to its pathogenesis [70]. Similarly, enhanced natural cytotoxicity receptors (NCRs) of NK cells in patients with HBV-ACLF are associated with disease progression [26]. IL-12- and IL-15-stimulated NK cells increase the secretion of TNF-*α* and IFN-*γ*. Furthermore, the stimulation of NK cells with IFN-*α* upregulates not only the expression of NKG2D but also the production of IFN-*γ*, perforin, TNF-*α*, and granzyme B. Blocking NKG2D resulted in partial downregulation of these cytokines, leading to impairment of NK function [71]. KCTD9 may also induce liver damage mediated by NK cell in HBV-ACLF [72]. The overexpression of KCTD9 results in significantly increased CD69 expression, enhanced cytotoxicity, and increased IFN-*γ* production. Inhibition of KCTD9 reduces the cytotoxic function of NK cells.

### 6.2. Adaptive Immune Cells in ACLF

Upregulation of T cell immunoglobulin and mucin domain-containing molecule-3 (Tim-3), CTLA-4, and PD-1 is found in adaptive immunocompromised T cells [73, 74]. And the ratio of regulatory T cells (Tregs) to T cells is higher in ACLF patients than that in normal subjects [75]. The decrease in ratio of CD3^+^ cells to monocytes (T/M) is associated with a poor prognosis of ACLF. The secretion of TNF-*α* in monocytes can be inhibited by CD4^+^ T cells, CD8^+^ T cells, and Treg, resulting in abnormal monocytes [76]. In addition, some studies have shown that patients with HBV-ACLF have significantly fewer CD4^+^ and CD8^+^ T cells compared with chronic hepatitis B (CHB) patients [77–79].

Besides, the diversity of CD8^+^ T cells of HBV-ACLF patients decreases during hospitalization, and the proportion of the top 100 CD8^+^ clonotypes increases. And the MELD score has a positive correlation with the diversity of CD8^+^ T cells and a negative correlation with the cumulative frequency of the top 100 clonotypes, suggesting that more CD8^+^ T cell expansion in the early stage is correlated with a better prognosis of HBV-ACLF patients [80].

## 7. Immunometabolism

Metabolism and immune regulation influence each other. On the one hand, the metabolism of immune cells changes from static state to active state in the process of immune response; on the other hand, the change of metabolism controls the differentiation and function of immune cells [81]. During systemic inflammatory responses, immune cells regulate cellular metabolism to meet high energy demands; the process of binding metabolism to immune cell responses is called immunometabolism [82].

Mitochondria are the centers of cell metabolism; it releases mitochondrial DNA (mtDNA), proteins, lipid metabolites, and ROS. These molecules can act as DAMPs that bind to PRRs to initiate an inflammatory response. There is a complex link between mitochondrial dysfunction and metabolic disorders in ACLF, resulting in reduced production of adenosine triphosphate (ATP), excessive storage of fat, and leakage of ROS [83, 84]. In the leukocytes of ACLF patients, mitochondrial dysfunction is represented by two breakpoints in the citric acid (TCA) cycle linked by an anaplastic reaction of glutaminolysis and nucleoside metabolism [85]. Among them, the metabolites of the TCA cycle can affect the differentiation of macrophages, and the low *α*-ketoglutarate/succinic acid ratio can enhance the activation of M1 macrophages. What is more, under the condition of inflammation, mitochondrial dysfunction leads to the enhancement of aerobic glycolysis and the increase of lactic acid as its end product [86]. Lactic acid produced by activated innate immune cells can limit inflammation and inhibit migration of monocytes and macrophages [87, 88].

Amino acid (AA) metabolism disorder plays an important role in immunometabolism of ACLF. Studies have shown that 43% of the 137 metabolites contained in the metabonomics database are associated with AAs, and the inflammatory response of ACLF patients is closely related to the change of AA metabolism [89]. Kynurenine promotes immune tolerance by inhibiting the proliferation of T cells and NK cells and promoting the proliferation of Tregs and MDSCs [90]. Arginine increases T cell oxygen consumption and mitochondrial respiration. L-glutamine, the most abundant extracellular amino acid, has also been shown to provide energy for T cell proliferation, cytokine secretion, and restoration of phagocytosis in monocytes [54, 91]. The glutamine synthase/glutaminase ratio in monocytes of ACLF patients is positively correlated with disease severity [54].

In addition, lipid metabolism disorder is closely related to ACLF. Inflammation is often associated with lipid metabolism disorders. Patients with ACLF have low high-density lipoprotein (HDL) particles and phospholipid content, especially lysophosphatidylcholine (LPC) containing omega-3 polyunsaturated fatty acid (PUFA), which is significantly decreased, but fatty acids are significantly increased [92–94]. Circulating saturated fatty acids can cause proinflammatory responses by enhancing the sensitivity of liver cells to TLR agonists [95, 96]. Excessive intake of linoleic acid (LA) leads to an increase in prostaglandin E2 (PGE2), which in turn inhibits macrophage secretion of proinflammatory cytokines and bacterial killing.

## 8. Microorganism and ACLF

In 40% of ACLF patients with cirrhosis without any identifiable precipitation conditions [8], the transferred bacteria or bacterial products may promote the occurrence and development of ACLF [97]. Impaired innate immune cell function in ACLF inhibits immune function and may lead to infection [47, 48]. In the late stages of ACLF, the proinflammatory response is further exacerbated by the increased probability of infection due to the development of CARS, which exacerbates liver injury [26]. SIRS and CARS are considered to be critical for effector functions of immune cells in ACLF (e.g., monocytes and macrophages), resulting in immune imbalance and bacterial translocation [98].

In addition, other changes also lead to increased bacterial translocation of intestinal microbiome to portal vein and lymphatic circulation in chronic liver disease and ACLF patients, such as altered composition of intestinal microbiome and increased intestinal permeability, which could lead to the permanent stimulation of the immune system by gut-derived PAMPs [99]. It is well known that in the setting of portal hypertension, increased shunting leads to bacterial escape in the reticuloendothelial system (RES) [100]. Therefore, portal hypertension is conducive to bacterial translocation, which is conducive to the activation of innate immunity [101, 102]. Systemic inflammation can also directly or indirectly increase bacterial translocation through enhancing circulatory dysfunction and stimulating sympathetic nervous system homeostasis [15, 103]. And the gut-liver axis acts as a bridge in bacterial translocation in ACLF.

### 8.1. Gut-Liver Axis

The gut-liver axis presents the immunomodulatory interactions between the gastrointestinal tract and the hepatic sinusoids, bidirectionally linked by the portal circulation and the biliary tree [104]. The liver receives portal blood rich in nutrients and pathogenic microbial products, which leads to moderate constant stimulation of antigens, and maintaining hepatic and systemic immune balance [3].

The microbiota, which includes bacteria, fungi, and viruses, is influenced by factors such as alcohol, diet (e.g., nonalcoholic fatty liver disease (NAFLD)), and drugs (e.g., antibiotics) [105–107]. The microbiota could also be altered by the use of proton pump inhibitors and repeated exposure to antibiotics [108]. The microbial community in cirrhosis shows significant reductions in bacterial diversity and in situ microbial communities. The changes of specific microbiota in cirrhosis mainly included the increase of *Fusobacteria*, *Proteobacteria*, *Enterococcaceae*, and *Streptococcaceae* and the relative decrease of *Bacteroidetes*, *Ruminococcus*, *Roseburia*, *Veillonellaceae*, and *Lachnospiraceae* [109]. The fecal microbial richness and species diversity of patients with decompensated cirrhosis are lower than those of patients with compensatory cirrhosis [110].

These changes in intestinal flora are due to reduced bile flow and cholestasis caused by cirrhosis, which impairs the enterohepatic circulation. This change may be more significant when accompanied by reduced intestinal motility, increased intestinal permeability, barrier dysfunction to lymphatic and hepatic sinusoid, portal hypertension, and immune system disorders [105, 106, 111]. Dysbiosis, in turn, can cause intestinal inflammation, disrupting gut barrier function, leading to bacterial translocations, and reducing the conversion of primary to secondary bile acids in the intestinal tract [112, 113]. Since bile acids are involved in the absorption of fats and fat-soluble proteins (e.g., vitamin K-dependent coagulation factors), they have a significant effect on metabolism and coagulation [109]. Bile acids can regulate the farnesoid X receptor axis and play an important role in the homeostasis of the epithelial barrier and the gut-vascular barrier [114, 115]. Bile acids can also modulate mucosal immune tolerance, controlling innate immune inflammatory signaling and adaptive immunity through regulating the ratio of Th17 and Treg cells, which is disordered in cirrhosis and liver failure [116, 117], while total serum bile acid is positively correlated with disease severity in patients with alcoholic hepatitis [118].

Besides, slow intestinal transport contributes to bacterial overgrowth, which in turn leads to bacterial ectopic development [108]. Short-chain fatty acids (SCFAs: butyric, acetic, and propionic acid) could decrease intraluminal pH, stimulate mucin production, increase intestinal motility, maintain intestinal epithelial cell viability, and keep enterocyte tight junction integrity [119]. In addition, SCFAs modulate immune responses in gut-associated lymphoid tissue (GALT). They inhibit activation of macrophages and DCs, induce the secretion of inflammatory cytokines, and form a pool of T helper cells [120]. Intestinal barrier disruption is related to downregulation of tight junction proteins occludin and Claudin-1 in intestinal epithelial cells [121].

What is more, gut-related immune system changes include reduced synthesis of antibacterial peptides, immunoglobulin A (IgA), defensins, and hypo- or achlorhydria, causing bacterial translocations that lead to decompensation of cirrhosis [109]. B lymphocytes in GALT play critical roles in luminal IgA secretion and defense against enteric pathogens and maintain gut microbial homeostasis by secreting commensal-specific IgA [122]. IgA in the intestinal lymphoid follicles is transferred to the intestinal lumen through endocytosis, thereby forming the microbiota [123].

## 9. Treatment

The main principle of ACLF treatment is the diagnosis and management of precipitating events, the most important precipitating events being infection and alcoholic hepatitis, and the general treatments include the application of vasoactive drugs, artificial liver support system (ALSS), and liver transplantation (LT). In addition, we also summarize the application of immunotherapy in ACLF, including targeting systemic inflammation, monocyte and macrophage, or intestinal flora. In addition, ACLF is mainly divided into two phases: early systemic inflammation (the first stage) is manifested as macrophage and DC activation, neutrophil activation, and T cell activation; late immunosuppression (the second stage) is characterized by the following: the hyporesponsive monocyte, decreased inflammatory cytokine production, interventions macrophage and neutrophil phagocytosis, and loss of Kupffer cells and T cells; exhibits Ab responses; and increased susceptibility to opportunistic infections [6]. Treatment may be different at these two different stages.

### 9.1. Treatment for Infection in ACLF

The most common inducing event in ACLF is infection, including bacterial, viral, and fungal infections. The prevalence of infection is approximately 50% in patients with ACLF, and slightly higher infection rates have been associated with an increased number of organ failures, a poorer outcome, and higher mortality [15, 124]. Bacterial infection control measures are therefore important in medical treatment [125]. A broad antibiotic regimen should cover all potential pathogens, with high-dose antibiotics administered within 48 and 72 hours of infection diagnosis to enhance clinical outcome and decrease selection of resistant strains [125]. Fluoroquinolone antibiotics have a clear therapeutic role in the prevention of spontaneous bacterial peritonitis [126]. The use of fluoroquinolones results in the expansion of Tregs and an improved proinflammatory milieu in the cirrhotic liver [127].

In addition to bacterial infections, patients with hepatitis B should be treated with potent antiviral drugs, such as tenofovir, tenofovir alafenamide, or entecavir [13], which can improve the prognosis of patients with HBV-ACLF [128–130]. What is more, one study reported that 43% of ACLF patients have invasive mycosis, which had higher mortality than patients negative for fungal infection [131, 132]. Invasive pulmonary aspergillosis (IPA) infection accounts for 5–8.3% of HBV-ACLF and 14% of severe alcoholic hepatitis (sAH) patients [133, 134]. Voriconazole is recommended as a first-line choice for IPA primary treatment, but due to its hepatotoxicity, its use in ACLF patients needed to be closely monitored, and its therapeutic trough concentration range of 1-5 *μ*g/mL should be maintained [135].

### 9.2. Treatment for Alcohol-Related ACLF

In western countries, active alcoholism and sAH contribute to alcohol-related ACLF [136]. One study recommends starting glucocorticoids in early-stage ACLF (the first stage) and early discontinuation of the drug before the onset of advanced immune paralysis (the second stage) [137]. Prednisolone, an anti-inflammatory corticosteroid, is widely recommended for sAH therapy [138, 139]. Glucocorticoid-mediated immunosuppression may increase the chance of bacterial infection with alcoholic hepatitis. Therefore, corticosteroids are best used for short periods of the first stage [140, 141].

However, the use of steroids in the later stages (the second stage) of ACLF, where the anti-inflammatory effect prevails, can be detrimental because it may increase susceptibility to infection [50]. For this reason, it is necessary to evaluate the efficacy of steroids with Lille score, which is based on age, total bilirubin levels, baseline creatinine levels, albumin levels, prothrombin time, and repeated total bilirubin levels [142, 143]. Patients can be classified as complete responders (≤0.16), partial responders (0.16–0.56), or nonresponders (>0.56) based on the Lille score to determine whether to continue or discontinue corticosteroid therapy [140]. Corticosteroid responses in ACLF patients are further reduced as the severity of ACLF increased. Patients with a Lille score of <0.45 had a poorer response to corticosteroids and had a lower 6-month survival rate [143].

What is more, some preliminary studies suggest that fecal microbiota transplantation from healthy donors also has beneficial effects on patients with alcohol-related ACLF [144, 145].

### 9.3. Other General Treatments for ACLF

Some vasoactive agents also play a role in ACLF therapy. Carvedilol improves survival rate and reduces acute kidney injury (AKI) and spontaneous bacterial peritonitis (SBP) events within 28 days in ACLF patients who have mild esophageal varices with a hepatic venous pressure gradient ≥ 12 mmHg [146]. In ACLF patients with hepatorenal syndrome-AKI, infusion of terlipressin has an earlier and higher response than treatment with norepinephrine, improving patient survival rate [147].

ALSS can remove toxic substances from the circulation through dialysis techniques [148] and improve the survival rate of HBV-ACLF patients compared with standard medical treatment [149]. Whole plasma exchange improves systemic inflammation and reduces the development of multiorgan failure in patients with ACLF and may be the preferred form of liver support in patients with ACLF [150].

ACLF patients who choose LT therapy have a better prognosis than ACLF patients who are ineligible or not selected for LT therapy [151], with 1-year survival rate approaching 80% after LT compared with less than 20% in patients who do not undergo LT [152–154]. Posttransplant survival in mild ACLF is similar to that in patients without ACLF [155].

### 9.4. Treatment of Systemic Inflammation (the First Stage) in ACLF

Because systemic inflammation plays a large role in ACLF, drugs targeting systemic inflammation are being explored. IL-22 treatment results in the reorganization of damaged regeneration pathways and protects cells from bacterial infection [45]. IL-22Fc could improve the survival of ACLF mice that enhance many antimicrobial genes via the antiapoptotic protein BCL2 [45].

In addition, stem cell transplantation clearly benefits HBV-ACLF patients through exerting a paracrine immune regulation effect [156]. Hepatocyte transplantation and treatment with bone marrow-derived stem cells, mesenchymal stem cells (MSCs), and multipotent mesenchymal stromal cells are called cell therapies. In two open-label controlled studies, ACLF patients are treated with MSCs or allogenic bone marrow MSCs [156, 157]. A study showed that MSCs have immunomodulatory and anti-inflammatory functions, which can alleviate liver inflammation, improve liver function and serum albumin, and decrease the chance of infection, which benefits survival rate. The 24-week survival rate is enhanced in the MSC group (73.2%) compared to the standard care group (55.6%) [14, 157].

Human serum albumin (HSA) is demonstrated to be a disease-modifying anti-inflammatory agent capable of reducing inflammatory cytokines in AD cirrhosis patients [97]. Albumin may also act intracellularly by internalizing into endocytic vesicles, thereby blocking inflammatory signaling pathways in ACLF [158]. Transcytosis caused by regulating albumin binding and endocytosis depends on at least seven endothelial and immune cell-associated proteins and receptors [159]. In addition to that, O'Brien et al. previously found that HSA can reverse PEG2-induced immune dysfunction [47].

Other immunomodulatory drugs are also being explored in ACLF [14]. TLR4 inhibitor TAK-242 improved survival rate in experimental ACLF-induced mice [160]. G-CSF can be used to treat hepatic encephalopathy, hepatorenal syndrome, and sepsis [161, 162]. Patients with ACLF had increased leukocyte and neutrophil counts and a decreased disease severity index after getting G-CSF treatment [161]. Moreover, studies have shown that pyroptosis plays an important role in the pathogenesis of ACLF. The pyroptosis of hepatocytes induced by high mobility group box-1 (HMGB1) amplifies the inflammatory response, thereby aggravating ACLF. Therefore, HMGB1 is also a potential target for ACLF therapy [163, 164].

### 9.5. Treatment of Targeting Monocyte and Macrophage

Monocyte and macrophage (including KCs) play a decisive role during the occurrence and development of ACLF; therefore, various abnormalities of them in ACLF can be targeted for treatment.

#### 9.5.1. Targeting Liver Macrophages

A study proposes that MERTK antagonism could be used in advanced ACLF (the second stage), when prolonged CARS is prone to infectious complications [48]. Addition of the MERTK inhibitor UNC569 restored response of monocytes to LPS [48]. Silencing HIF1A-AS1 decreased KC apoptosis induced by TNF-*α*, which also provides a molecular basis for the ACLF therapy [56].

More importantly, the high scavenging capacity of liver macrophages, especially KCs, enables them to be preferentially targeted by drug carrier materials, including hard shell microvesicles, polymers, and liposomes [165], which helps these carrier materials to play a more direct role in controlling liver inflammation.

#### 9.5.2. Inhibiting Activation of Kupffer Cell

In early stages of ACLF (the first stage), limiting profound innate immune activation is a useful immunotherapy strategy. The initial inflammation is primarily mediated by KCs. Then, NF-*κ*B signaling pathways and the P3 inflammasome are also activated, which can be targets of immunotherapy. Another strategy is to target DAMPs, including HMGB-1 and histones [5]. Affecting the gut barrier or gut microbiota, the use of probiotics or antibiotics may alleviate KC activation [166].

#### 9.5.3. Inhibiting Recruitment of Monocyte to the Liver

Monocytes expand in liver disease. In animal models and patients, they are driven by chemokine-chemokine receptor interactions, such as CCL2/CCR2- and CCL5/CCR5-mediated pathways which have a primary function in ACLF [48, 167]. This leads to ideas for targeted therapies, for example, with monoclonal antibodies or small molecule inhibitors [168]. Interestingly, TLR-3 agonists are worthy of further study as potential drugs [55].

### 9.6. Treatment of Targeting Immunometabolism

By feeding glutamine or *α*-ketoglutarate into the TCA cycle, the phagocytosis of monocytes in ACLF patients could be restored [54, 169]. Succinic acid is also a potential therapeutic target for patients with ACLF [169]. Peroxisome proliferator-activated receptor *γ* coactivator 1*α* (PGC-1*α*), as a major regulator of mitochondria, is a potential therapeutic target for improving mitochondrial dysfunction [170]. To restore lipid metabolism balance, HDL can be increased or the liver X receptor (LXR) can be activated [171, 172].

### 9.7. Treatments Targeting Gut Microbiota

Probiotics are live microorganisms that, when ingested, provide benefits to the host, either directly or indirectly. It has been shown that they are effective in SCFA production, intestinal barrier integrity, alterations in colonic pH, and modulation of the immune system [173]. Obeticholic acid can reduce portal hypertension and improve intestinal flora, which may have potential benefits in ACLF patients [174]. Long-term oral administration of poorly absorbed antibiotic to prevent bacterial translocation can prevent major cirrhosis complications such as hepatic encephalopathy and AKI [175–177]. Recent translational studies suggest that defective farnesoid X receptor signaling plays a critical role in liver inflammation, intestinal bacterial translocation, and portal hypertension, promoting inflammation in ACLF, and may be targeted by drug agonists [7]. Fecal microbial transplantation (FMT) has been tested in primary sclerosing cholangitis and has been shown to be safe and closely related with significantly increased biodiversity and improved alkaline phosphatase activity. In an animal model of diet-induced nonalcoholic steatohepatitis (NASH), normalization of the gut microbiota could directly reverse portal hypertension [178].

## 10. Transcriptomics and ACLF

### 10.1. PBMC Transcriptomics in HBV-ACLF

Compared with alcoholic liver disease-associated ACLF patients, abnormal immune processes are more pronounced in HBV-ACLF patients [24]. Transcriptome analysis of HBV-ACLF suggests that viral, immune, and metabolic processes play a central role in the biological process network from acute-on-chronic hepatic dysfunction (ACHD) to ACLF. Virus correlation analysis suggests that virus is involved in various stages of HBV-ACLF. Analysis of immune-related blood transcriptional module (BTM) shows that the expression of genes associated with innate immune response is significantly upregulated while that of genes associated with adaptive immune response (T cells, B cells, and NK cells) is downregulated; as a result, adaptive immunity of ACLF patients may be exhausted [179].

The differentially expressed genes (DEGs) associated with innate immune responses are most significant in five modules (interferon, monocytes, neutrophils, inflammation, and dendritic cells). In the interferon module, including antiviral interferon signature, innate antiviral response and type I interferon response are significantly upregulated. The expression level of these DEGs is the highest in the ACHD group and significantly downregulated in the ACLF group, suggesting that the interferon module may be the initial factor for the occurrence of ACLF. Compared with the normal control (NC) group, monocyte module genes in the ACHD and ACLF groups are significantly upregulated and BTMs associated with neutrophils, inflammatory, dendritic cells, and antigen presentation show consistent changes in the ACHD and ACLF groups [179].

Compared with other diseases, the increased expression of metabolic genes in ACLF patients, most notably genes in PPAR and mTOR signaling pathways, further suggests that lipid metabolic disorders may play a key role in the development of ACLF. Compared with the NC group, the expressions of thrombospondin 1 (THBS1), MERTK, semaphorin 6B (SEMA6B), and PPAR *γ* (PPARG) genes are significantly increased in the ACLF group, and these four genes are closely related to innate immune response, adaptive immune response, complement activation, fatty acid oxidation, and reactive oxygen species metabolism, suggesting a virus-based immune metabolic disorder [179].

### 10.2. Prospects of Application of Single-Cell RNA Sequencing in ACLF

While transcriptomics can assess immunity and metabolism during ACLF, it may obscure the key contribution of individual cell populations, while single-cell RNA sequencing can understand the role of single-cell population in ACLF by classifying each cell into different subpopulations based on DEGs.

The application of single-cell RNA sequencing technology in a variety of diseases is conducive to a deeper understanding of diseases and more effective treatment. Its application in the liver can provide a framework for understanding the cellular basis of human liver functions and diseases [180]. It has revealed different subsets of liver nonparenchymal cells and highly specific gene expression pathways in liver fibrosis, NAFLD, and other pathological states, which is helpful to better guide the diagnosis and treatment of the liver diseases [181]. For example, it shows a new TREM2^+^CD9^+^ macrophage subpopulation in human cirrhosis, refines the definition of endothelial subsets, and proposes new therapeutic targets for cirrhosis [182]. It also shows that alpha-smooth muscle actin, a key marker of HSC activation, is only present in a subset of activated myofibroblasts, and S100A6 is a novel universal marker of activated myofibroblasts in liver fibrosis [183]. In addition, Trem2^+^ macrophages are termed “NASH-associated macrophages” by single-cell RNA sequencing technology in NASH mice [184]. The proportion of cells in chronic liver disease can also be measured using single-cell RNA sequencing data from healthy liver and peripheral immune cells, and the role of different cell types in each liver disease can be predicted based on large amounts of RNA sequencing data [185].

By application of single-cell RNA sequencing technology in acute liver failure (ALF) mice, it shows that new subpopulations of stellate cells, endothelial cells, KCs, monocytes, and neutrophils, along with their complex intercellular interactions, contribute to the development of ALF. The hepatic stellate cells are divided into four different populations (Lrat^high^ quiescent, Col1a1-positive fibrotic, Acta2-positive ALF-activated, and cycling stellate cells); the endothelial cell population is defined as three subpopulations and ALF-activated endothelial cells; Kupffer cells are identified as one quiescent and one ALF-activated population. Gene Ontology enrichment analysis of ALF-activated stellate cells finds terms associated with cell death and upregulated genes (*Trp53*, *Cdkn1a*, *Timp1*, and *Ereg*), which triggers cell cycle arrest and senescence. Gene Ontology term enrichment analysis of upregulated genes in ALF-activated endothelial cells reveals terms associated with gene expression and vascular remodeling. Gene Ontology enrichment analysis of ALF-activated Kupffer cells reveals terms about chemotaxis, cell migration, immune response, and apoptosis. Two populations of Ly6C-positive monocytes are identified, and the gene expression heterogeneity in the main population reveals that one consists of monocyte homing to the liver and the other induction of MHCII complex gene expression. Two neutrophil subpopulations are found; the smaller group is the proinflammatory subtype, participating in the regulation of the antioxidant transcriptional program. It also reveals that the activation of stellate cells, endothelial cells, and KCs during ALF is related with a common MYC-dependent transcription program, which is regulated by the gut microbiome through Toll-like receptor (TLR) signaling. In humans, it shows that the MYC expression is upregulated in the liver of ALF transplant recipients compared to healthy donors [186].

Currently, single-cell RNA sequencing is beginning to be used in ACLF, it can be used to classify various immune cells in ACLF to study the role of various immune cell subpopulations in the occurrence and development of ACLF, and single-cell RNA sequencing reveals a panel of apoptotic and dysfunctional lymphatic endothelial cells as a result of secreted phosphoprotein 1 released by infiltrating monocytes/macrophages, which is closely related to the significant reduction of intrahepatic lymphatic vessels in HBV-ACLF patients [187]. However, this is only the beginning, as the sample size of ACLF increases and the incorporation of single-cell RNA sequencing into other functional assays will greatly improve our understanding of ACLF.

## 11. Prognosis

The prognosis of ACLF is closely related to infection and severity. ACLF can be divided into three grades according to the number of organ failure: ACLF-1 represents single renal failure or single nonrenal organ failure, ACLF-2 represents failure of two organs, and ACLF-3 represents failure of three to six organs [188]. The survival rate of mild ACLF, including ACLF-1 and ACLF-2, decreases significantly with infection, while infection has no significant effect on the prognosis of severe ACLF, such as ACLF-3 [15].

There are also many prognostic predictors of ACLF. The P5 score, a prognostic score that includes plasminogen levels, the incidence of hepatic encephalopathy, age, the international normalized ratio, and total bilirubin, is a promising prognostic score for HBV-ACLF [189]. In addition, macrophage secretions, such as sCD163 and macrophage inflammatory protein 3*α*, can be used as prognostic indicators of ACLF [190, 191]. What is more, some odd type predictors, such as neutrophil/lymphocyte ratio (NLR), can also predict ACLF mortality [192]. NLR scores of ≥3 are associated with low mortality; however, NLR scores of >6 are associated with higher mortality [26].

## 12. Conclusion

**Figure 1:**
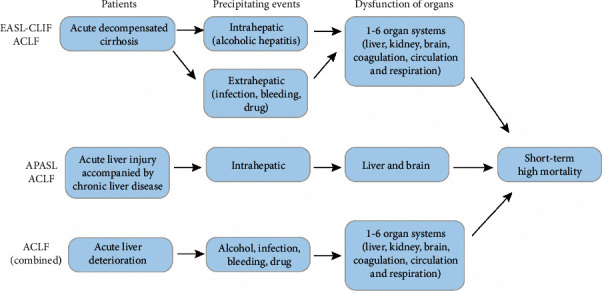
The clinical features of ACLF. Combining the definitions of EASL-CLIF ACLF and APASL ACLF, we defined ACLF as a syndrome of acute liver deterioration induced by precipitating events in patients with cirrhosis or noncirrhotic chronic liver disease, leading to failure of two or more organs, accompanied by short-term high mortality.

**Figure 2:**
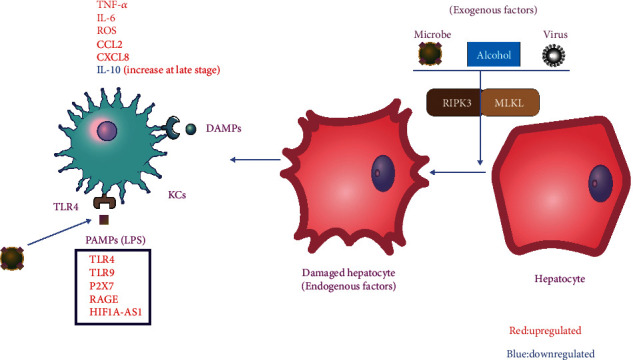
The initiation of hepatic inflammation. Bacteria, HBV, alcohol, and other external factors lead to hepatocyte necrosis through regulatory proteins such as RIPK3 and MLKL, and DAMPs produced by them are recognized by DAMP receptors such as TLR4, TLR9, P2X7, and RAGE expressed by macrophages/KCs. Or PAMPs produced by various pathogens, recognized by PRRs expressed by macrophages/KCs. Macrophages/KCs can secrete reactive oxygen species (ROS), proinflammatory cytokines, and chemokines; enhance inflammatory signals; and recruit other immune cells to the liver, speeding up the inflammatory process.

**Figure 3:**
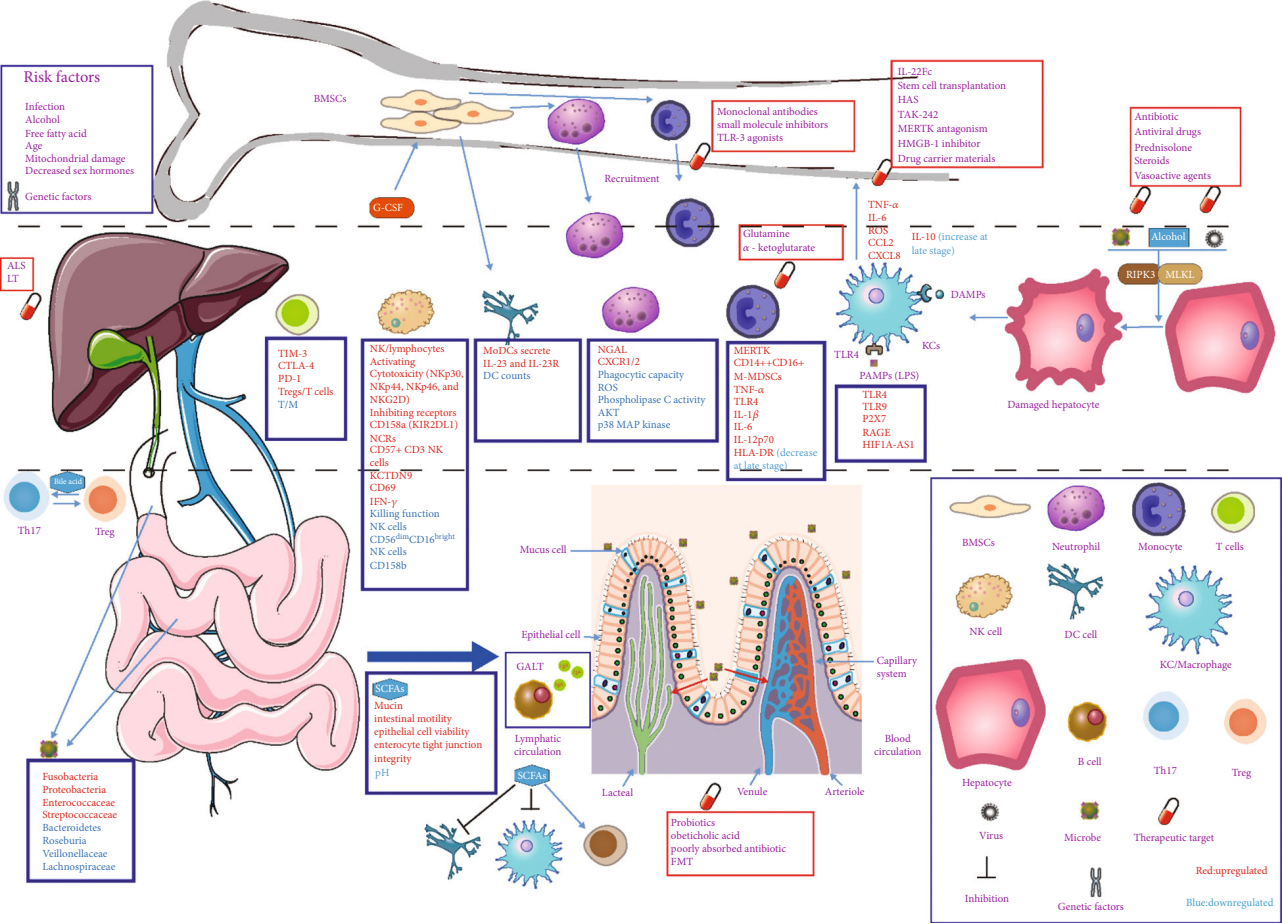
Mechanism by which the immune system and microorganisms jointly promote ACLF formation. DAMPs produced by damaged liver cells or PAMPs produced by pathogens are recognized by PRRs on macrophages/KCs, which are activated and release inflammatory factors and chemokines, and recruit neutrophils and monocytes. Along with BMSCs, DCs are activated, and NK cells, T cells, and B cells are consumed, inducing systemic inflammation. Various changes of cell surface factor and regulatory factor, immune metabolic disorders, and liver bile secretion decreased; regulation of TH17/Treg obstacle, late performance for immunosuppression and abnormal lipid metabolism, and the role of short-chain fatty acids regulate intestinal function decline, the intestinal barrier damage, and gut bacteria translocation, aggravating infection, and liver cell damage are aggravating, forming a vicious cycle.

**Figure 4:**
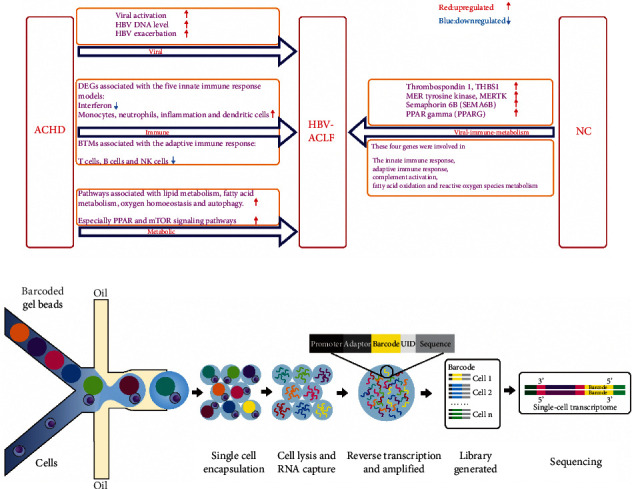
(a) PBMC transcriptomics in the HBV-ACLF expression. (b) Technology of single-cell RNA sequencing.

ACLF is a syndrome of acute liver deterioration induced by precipitating events (especially alcoholic and hepatitis B) in patients with cirrhosis or noncirrhotic chronic liver disease, leading to failure of two or more organs, accompanied by short-term high mortality (Figure 1). ACLF is usually initiated by hepatic inflammation (Figure 2). The persistence of hepatic inflammation can develop into systemic inflammation, and macrophages play a leading role in this process. They could secrete a large number of cytokines and recruit various immune cells, leading to liver and systemic inflammation. Among the mediators of inflammation, the proinflammatory cytokines (TNF-*α*, IL-1*β*, and IL-6) are involved in the occurrence of liver injury, while G-CSF improves liver function and survival in ACLF. As the disease progresses, it is also associated with immunosuppressive and some phenotypic changes of immune cells that play a major role in the process; for example, monocyte and macrophage show a decrease in the HLA-DR expression and an increase in the MERTK expression. What is more, during the development of ACLF, immunometabolism (mitochondrial dysfunction, AAs, and lipid metabolism disorder) and microorganisms (bacterial translocation and gut-liver axis) play an important role in it. Based on the detailed study of the development of ACLF, we summarize the general treatment and immunotherapy for different stages of ACLF (Figure 3). PBMC transcriptome analysis of HBV-ACLF suggests that viral, immune, and metabolic processes play a central role in the biological process network from ACHD to ACLF. And single-cell RNA sequencing reveals a panel of apoptotic and dysfunctional lymphatic endothelial cells as a result of secreted phosphoprotein 1 released by infiltrating monocytes/macrophages in HBV-ACLF patients, and we prospect further application of single-cell RNA sequencing in ACLF (Figure 4). Finally, the prognosis of ACLF is closely related to infection and severity, and the P5 score can be used to predict the prognosis of ACLF.

## Data Availability

No underlying data was collected or produced in this study.

## Abbreviations

ACLF: Acute-on-chronic liver failure
KCs: Kupffer cells
PAMPs: Pathogen-associated molecular patterns
DAMPs: Damage-associated molecular patterns
PRRs: Pattern recognition receptors
DCs: Dendritic cells
HBV-ACLF: Hepatitis B virus-associated ACLF
Tregs: Regulatory T cells
CHB: Chronic hepatitis B
EASL-CLIF: European Association for the Study of the Liver-Chronic Liver Failure Consortium
APASL: Asian Pacific Association for the Study of the Liver
AD: Acute decompensation
PBMCs: Peripheral blood mononuclear cells
LPS: Lipopolysaccharide
SIRS: The systemic inflammatory response syndrome
CARS: Compensatory anti-inflammatory responses
TLR: Toll-like receptor
ROS: Reactive oxygen species
HSECs: Hepatic sinusoidal endothelial cells
MoMFs: Monocyte-derived macrophages
GM-CSF: Granulocyte-macrophage colony-stimulating factor
G-CSF: Granulocyte colony-stimulating factor
mDCs, pDCs: Myeloid and plasmacytoid DCs
Tim-3: T cell immunoglobulin and mucin domain-containing molecule-3
RES: Reticuloendothelial system
MERTK: MER tyrosine kinase
M-MDSCs: Monocytic myeloid-derived suppressor cells
HIF1A-AS1: Hypoxia-inducible factor 1 alpha-antisense RNA 1
NGAL: Neutrophil gelatinase-associated lipocalin
fMLP: fMet-Leu-Phe
MoDCs: Monocyte-derived dendritic cells
NCRs: Natural cytotoxicity receptors
T/M: CD3^+^ cells to monocytes
mtDNA: Mitochondrial DNA
ATP: Adenosine triphosphate
TCA: Citric acid
AAs: Amino acids
HDL: High-density lipoprotein
LPC: Lysophosphatidylcholine
PUFA: Polyunsaturated fatty acid
LA: Linoleic acid
PGE2: Prostaglandin E2
NAFLD: Nonalcoholic fatty liver disease
SCFAs: Short-chain fatty acids
GALT: Gut-associated lymphoid tissue
IgA: Immunoglobulin A
ALSS: Artificial liver support system
LT: Liver transplantation
IPA: Invasive pulmonary aspergillosis
sAH: Severe alcoholic hepatitis
AKI: Acute kidney injury
SBP: Spontaneous bacterial peritonitis
MSCs: Mesenchymal stem cells
HSA: Human serum albumin
HMGB1: High mobility group box-1
PGC-1*α*: Peroxisome proliferator-activated receptor *γ* coactivator 1*α*
LXR: The liver X receptor
FMT: Fecal microbial transplantation
NASH: Nonalcoholic steatohepatitis
ACHD: Acute-on-chronic hepatic dysfunction
BTM: Blood transcriptional module
DEGs: Differentially expressed genes
NC: Normal controls
THBS1: Thrombospondin 1
SEMA6B: Semaphorin 6B
PPARG: PPAR *γ*
ALF: Acute liver failure
NLR: Neutrophil/lymphocyte ratio.
